# Effect of Arginase Inhibition on Ischemia-Reperfusion Injury in Patients with Coronary Artery Disease with and without Diabetes Mellitus

**DOI:** 10.1371/journal.pone.0103260

**Published:** 2014-07-29

**Authors:** Oskar Kövamees, Alexey Shemyakin, John Pernow

**Affiliations:** Department of Medicine, Karolinska Institutet, Stockholm, Sweden; University of Pecs Medical School, Hungary

## Abstract

**Background:**

Arginase competes with nitric oxide synthase for their common substrate L-arginine. Up-regulation of arginase in coronary artery disease (CAD) and diabetes mellitus may reduce nitric oxide bioavailability contributing to endothelial dysfunction and ischemia-reperfusion injury. Arginase inhibition reduces infarct size in animal models. Therefore the aim of the current study was to investigate if arginase inhibition protects from endothelial dysfunction induced by ischemia-reperfusion in patients with CAD with or without type 2 diabetes (Clinical trial registration number: NCT02009527).

**Methods:**

Male patients with CAD (n = 12) or CAD + type 2 diabetes (n = 12), were included in this cross-over study with blinded evaluation. Endothelium-dependent vasodilatation was assessed by flow-mediated dilatation (FMD) of the radial artery before and after 20 min ischemia-reperfusion during intra-arterial infusion of the arginase inhibitor (N^ω^-hydroxy-nor-L-arginine, 0.1 mg/min) or saline.

**Results:**

The forearm ischemia-reperfusion was well tolerated. Endothelium-independent vasodilatation was assessed by sublingual nitroglycerin. Ischemia-reperfusion decreased FMD in patients with CAD from 12.7±5.2% to 7.9±4.0% during saline administration (P<0.05). N^ω^-hydroxy-nor-L-arginine administration prevented the decrease in FMD in the CAD group (10.3±4.3% at baseline vs. 11.5±3.6% at reperfusion). Ischemia-reperfusion did not significantly reduce FMD in patients with CAD + type 2 diabetes. However, FMD at reperfusion was higher following nor-NOHA than following saline administration in both groups (P<0.01). Endothelium-independent vasodilatation did not differ between the occasions.

**Conclusions:**

Inhibition of arginase protects against endothelial dysfunction caused by ischemia-reperfusion in patients with CAD. Arginase inhibition may thereby be a promising therapeutic strategy in the treatment of ischemia-reperfusion injury.

## Introduction

Current treatment strategy for ST-elevation myocardial infarction involves rapid reperfusion of the occluded coronary artery by percutaneous coronary intervention or thrombolysis to limit infarct size [1]. However, rapid restoration of blood flow to the ischemic tissue itself may cause additional damage to the myocardium and coronary vasculature referred to as reperfusion injury [2]. The mechanism(s) behind reperfusion injury is complex and include oxidative stress, intracellular calcium ion overload, inflammation and opening of ion channels in the mitochondrion [3]. Endothelial dysfunction, characterized by reduced bioavailability of nitric oxide (NO), is known to be central for the development of reperfusion injury [4], [5]. The protection mediated by NO in the setting of ischemia-reperfusion include among several effects increased microvascular perfusion due to vasodilatation, anti-inflammatory effects and scavenging of reactive oxygen species [6]. NO may in addition prevent calcium release from the sarcoplasmatic reticulum and activation of mitochondrial permeability transition pores which is a central process in reperfusion injury [2]. Accordingly, restoration of NO bioavailability has been shown to reduce the extent of reperfusion injury in experimental studies via a mechanism related to anti-inflammatory effects, scavenging of reactive oxygen species and preservation of mitochondria function [6]–[8].

Arginase is a metalloprotease that has emerged as an important regulator of NO production throughout the cardiovascular system [9]. A delicate balance exists between arginase and NO synthase (NOS) by their competition for the common substrate L-arginine [9]. Up-regulation of arginase leads to impaired NO production, increased levels of reactive oxygen species and induction of endothelial dysfunction [10]. Arginase has therefore been suggested to play an important role in reperfusion injury [11]. Accordingly, inhibition of arginase markedly reduces infarct size in animal models of ischemia-reperfusion via a mechanism involving preserved bioavailability of NO [12]–[14]. The role of arginase in the development of ischemia-reperfusion injury in patients with coronary artery disease (CAD) has previously not been investigated, however. Based on the experimental data described above, we hypothesized that arginase is of pathophysiological importance in ischemia-reperfusion and that inhibition of arginase activity will prevent the development of ischemia-reperfusion injury in patients with CAD.

We have recently demonstrated that arginase inhibition improves basal endothelial function in patients with CAD with or without concomitant type 2 diabetes mellitus [15]. The improvement in endothelial function was greater in patients with diabetes than in patients without diabetes suggesting that the role of arginase may be particularly important in patients with type 2 diabetes. Available data suggest that treatment strategies to prevent ischemia-reperfusion injury may be less effective in the presence of type 2 diabetes [16]. Accordingly, it appears to be of importance to develop effective therapies that improves endothelial function following ischemia-reperfusion in patients with a common co-morbidity such as type 2 diabetes. Therefore, we investigated the effect of local arginase inhibition on endothelial dysfunction induced by ischemia- reperfusion in the forearm of patients with CAD with and without type 2 diabetes.

## Methods

### Subjects

Patients were recruited from the coronary angiography and angioplasty registry at Karolinska University Hospital between December 2011 and June 2013. One group (n = 12) consisted of patients with CAD and the other group (n = 12) consisted of patients with CAD and type 2 diabetes mellitus (CAD+DM). Patients with CAD defined as previous myocardial infarction or as ≥50% stenosis in one or more of the coronary arteries during angiography treated with percutaneous coronary intervention or coronary artery bypass grafting were eligible for the study. Type 2 diabetes was defined as fasting blood glucose exceeding 7 mmol/l on two different occasions, blood glucose exceeding 11 mmol/l two hours after oral administration of 75 g of glucose or medical history of type 2 diabetes. Absence of type 2 diabetes in the CAD group was verified by an oral glucose tolerance test. Exclusion criteria were age >80 years, acute myocardial infarction or unstable angina within 3 months prior to the experiment, presence of Raynaud's phenomena, arterio-venous shunt or other vessel abnormality in the forearm, any condition that interfered with the probability of completing the study protocol, participation in another study or unwillingness to participate. Patient characteristics are summarized in Table 1. The study was conducted according to the principles outlined by the declaration of Helsinki and was approved by the regional ethical review board in Stockholm. All patients were informed of the purpose and possible risks of the study and gave their oral and written informed consent. Clinical trial registration number: NCT02009527.

**Table 1:** Basal characteristics.

	CAD	CAD+DM
Age (years)	65±8	65±7
Blood pressure (mmHg)		
Systolic	131±15	141±12
Diastolic	80±9	83±10
MAP	97±10	102±9
BMI (weight/height^2^)	28±4	27±3
Waist-hip ratio	0.99±0.06	0.98±0.06
HbA1c (%)	39±3	52±8*
Glucose (mmol/l)	5.6±0.8	8.0±2.2†
Triglycerides (mmol/l)	1.3±0.8	1.9±1.0
Total cholesterol (mmol/l)	4.3±0.8	4.2±1.1
HDL cholesterol (mmol/l)	1.3±0.3	1.1±0.3
LDL cholesterol (mmol/l)	2.4±0.5	2.2±0.8
**Medication (no.)**		
ACE-inhibitors or ARB	8	10
Beta-blockers	11	8
Platelet inhibitors	12	12
Lipid lowering drugs	11	10
Diuretics	2	3
Nitrates	1	4
Oral glucose lowering drugs	0	8
Insulin	0	4
Calcium channel blockers	0	3

Data are presented as mean and SD except medication which is presented as numbers. ARB, angiotensin receptor blocker; ASA, acetylsalicylic acid; BMI, body mass index; CAD, coronary artery disease; DM, type 2 diabetes mellitus; MAP, mean arterial pressure; HbA1c, glycated hemoglobin; HDL, high density lipoproteins; LDL, low density lipoproteins.

*P<0.001.

† P<0.01.

### Flow-mediated vasodilatation

Non-invasive examination of the radial artery of the non-dominant arm (left arm for all subjects) was performed with a 11 MHz (output 12 MHz) linear-array transducer connected to a Vivid E9® (GE, Waukesha, Wisconsin, USA). The transducer was connected to a flexible tripod to prevent movement of the probe. Images were recorded and saved every third second at end-diastole. Baseline radial artery diameter was recorded for one minute and defined as a mean from 20 images. The radial artery was used for determination of flow-mediated dilatation (FMD) since the brachial artery was catheterized for study drug infusions (see below). A blood pressure cuff was placed around the upper part of the forearm, which was inflated to 30 mmHg above systolic pressure or 200 mmHg for 5 min. The diameter of the radial artery was continuously recorded for 3 min during hyperemia induced by deflation of the cuff. The three frames displaying maximum dilatation at end-diastole (triggered from the ECG) were used to calculate a mean diameter [17]. All images were analyzed with Brachial analyzer (Medical Imaging Applications, Iowa City, IA, USA). FMD was calculated as a percentage increase in diameter from baseline diameter according to the following formula: diameter following cuff deflation-baseline diameter/baseline diameter x 100. The coefficient of variation is 18% [18].

### Study protocol

**Figure 1:**
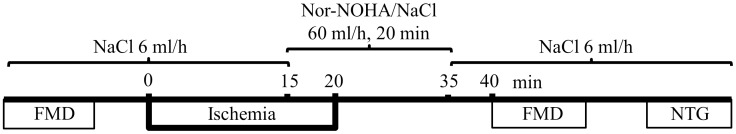
Study protocol. FMD, flow-mediated dilatation; NaCl, sodium chloride; Nor-NOHA, N^ω^-hydroxy-nor-arginine; NTG, nitroglycerine.

The design of the study was randomized cross-over with blinded evaluation. One single operator who was unaware of the intervention performed all FMD registrations, analyses and calculations. Subjects performed the protocol twice, on one occasion active treatment was administrated and on the other placebo (saline). The order of how the subjects received intervention was randomly assigned by drawing one of two numbers written on two pieces of paper. The experiment was implemented in a dimly lit room in a calm environment. The subject did not take their morning medication and were instructed to refrain from caffeine-containing drinks or nicotine product on the day of the study. A light standardized breakfast was provided to the subjects before the experiments. A percutaneous catheter was introduced in the brachial artery of the non-dominant arm (left arm for all subjects) in the proximal direction following local infiltration of anesthesia with 2 ml carbocaine (10 mg/ml, AstraZeneca, Södertälje, Sweden). The arm was then fixed with a vacuum cushion to prevent excessive movement during the FMD. An i.a. infusion of saline (0.9%) was started at rate at 6 ml/h. The experimental protocol is schematically illustrated in Fig. 1. Baseline endothelium-dependent vasodilatation was determined with FMD, after which whole arm ischemia was induced for 20 min by inflation of a blood pressure cuff placed on the upper arm as described previously [19], [20]. Reperfusion was initiated by deflation of the upper arm cuff. At 15 min of ischemia an i.a. infusion of either saline or the arginase inhibitor N^ω^-hydroxy-nor-arginine (nor-NOHA, Bachem, Bubendorf, Switzerland) was started at a dose of 0.1 mg/min (60 ml/h) and continued for 20 min i.e. until 15 min of reperfusion. FMD was re-evaluated at 20 min of reperfusion i.e. five min after the infusion of saline or nor-NOHA was stopped. The endothelium-independent vasodilatation was assessed at the end of the experiment by applying sublingual nitroglycerine (0.4 mg, PharmaPol, Dägeling, Germany). The diameter of the radial artery was determined for 5 min and the maximum increase was recorded. Nitroglycerine was not administered before ischemia due to that its long-acting vasodilatation may influence baseline radial artery diameter following ischemia. Exogenous administration of NO donors is not affected by ischemia-reperfusion [21]. Therefore, only one time point at the end of the protocol was used to evaluate the effect of arginase inhibition on endothelium-independent vasodilatation. Nor-NOHA was dissolved in double-distilled water, sterile filtrated through a Millipore filter, tested for bacterial toxins and sterility and stored frozen at −80°C.

### Blood sampling

Fasting venous blood samples were collected on the first study occasion at fasting condition for analysis of low density lipoprotein, high density lipoprotein and total cholesterol, triglycerides, glucose and glycated hemoglobin (HbA1c).

### Calculation and statistics

Based on the different outcome following acute myocardial infarction depending on the presence and absence of diabetes [22], [23] and the different response to arginase inhibition on baseline endothelial function in patients with and without type 2 diabetes these two groups were analyzed separately [15]. Data are presented as means and SD. Based on an absolute improvement of FMD by 3% and a SD of 1.85% [24], the number of subjects needed to reach 80% power with a 2-sided test with a significance level of 5% was calculated to be 12. The study was designed to analyze the change in FMD separately in the CAD group and the CAD+DM group. In addition to that, pooled data from both groups was analyzed. Basal characteristics and endothelium-independent vasodilatation was analyzed with paired t-test. 2-way ANOVA with multiple comparison and correction for multiple analyses with Sidak test was used to analyze FMD. Radial artery diameter within each group of patients was analyzed with 1-way ANOVA.

## Results

### Basal characteristics

Basal clinical characteristics are presented in Table 1. Groups were matched for age. Blood pressure did not differ significantly between the groups, although patients belonging to the CAD+DM group tended to have a higher systolic blood pressure. Patients in the CAD+DM group had significantly higher levels of fasting glucose and HbA1c. The experimental protocol was well tolerated by all subjects. The subjects felt a numb sensation in the forearm during ischemia but no patient experienced any pain. No adverse effects of the infusions were noticed.

### Endothelium-dependent and endothelium-independent vasodilatation

**Figure 2:**
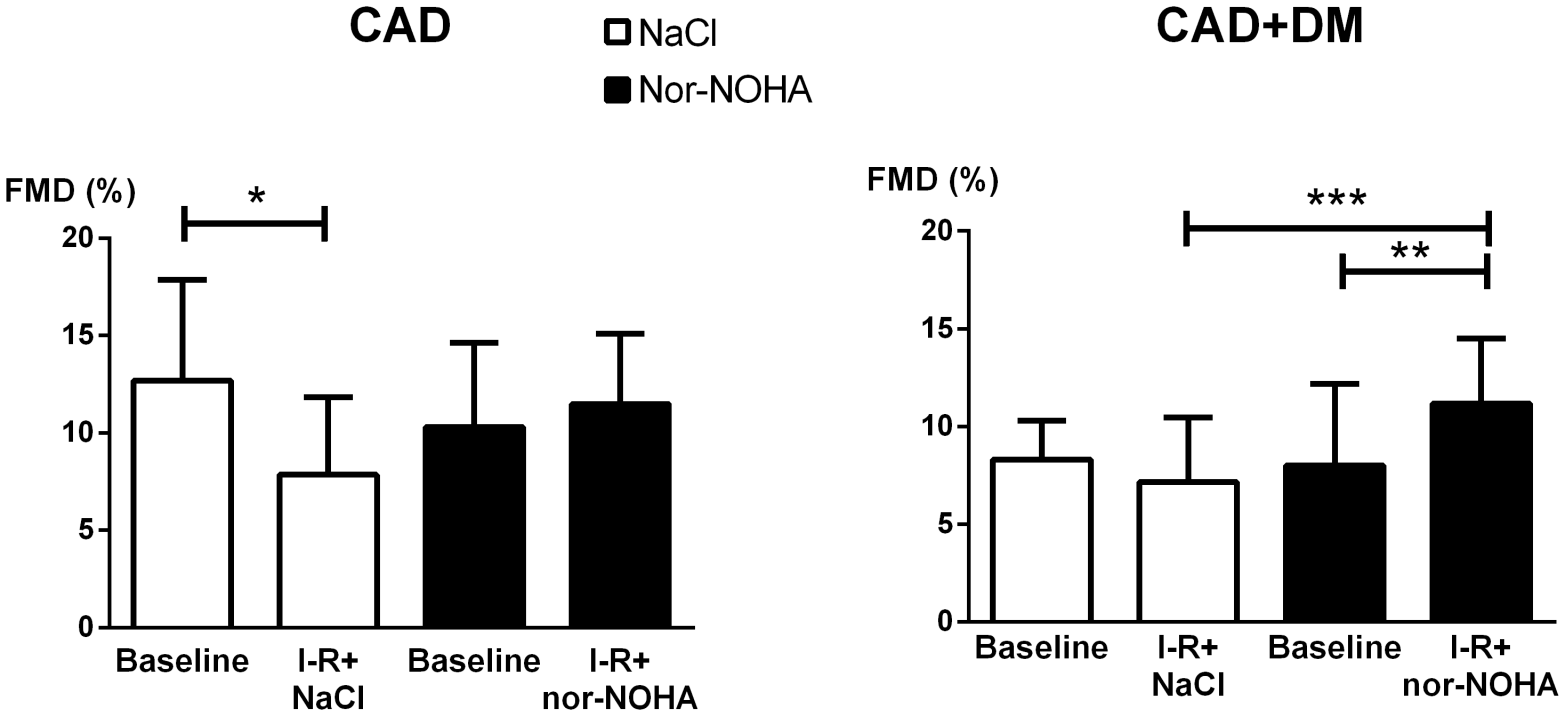
Flow-mediated dilatation of the radial artery for patients with CAD and CAD+DM respectively. Flow-mediated dilatation (FMD) of the radial artery at baseline and after ischemia-reperfusion (I-R) in patients with coronary artery disease (CAD) and CAD plus type 2 diabetes mellitus (CAD+DM) following administration of saline or the arginase inhibitor N^ω^-hydroxy-nor-arginine (nor-NOHA). Data are presented as means and SD; *P<0.05, **P<0.01.

**Figure 3:**
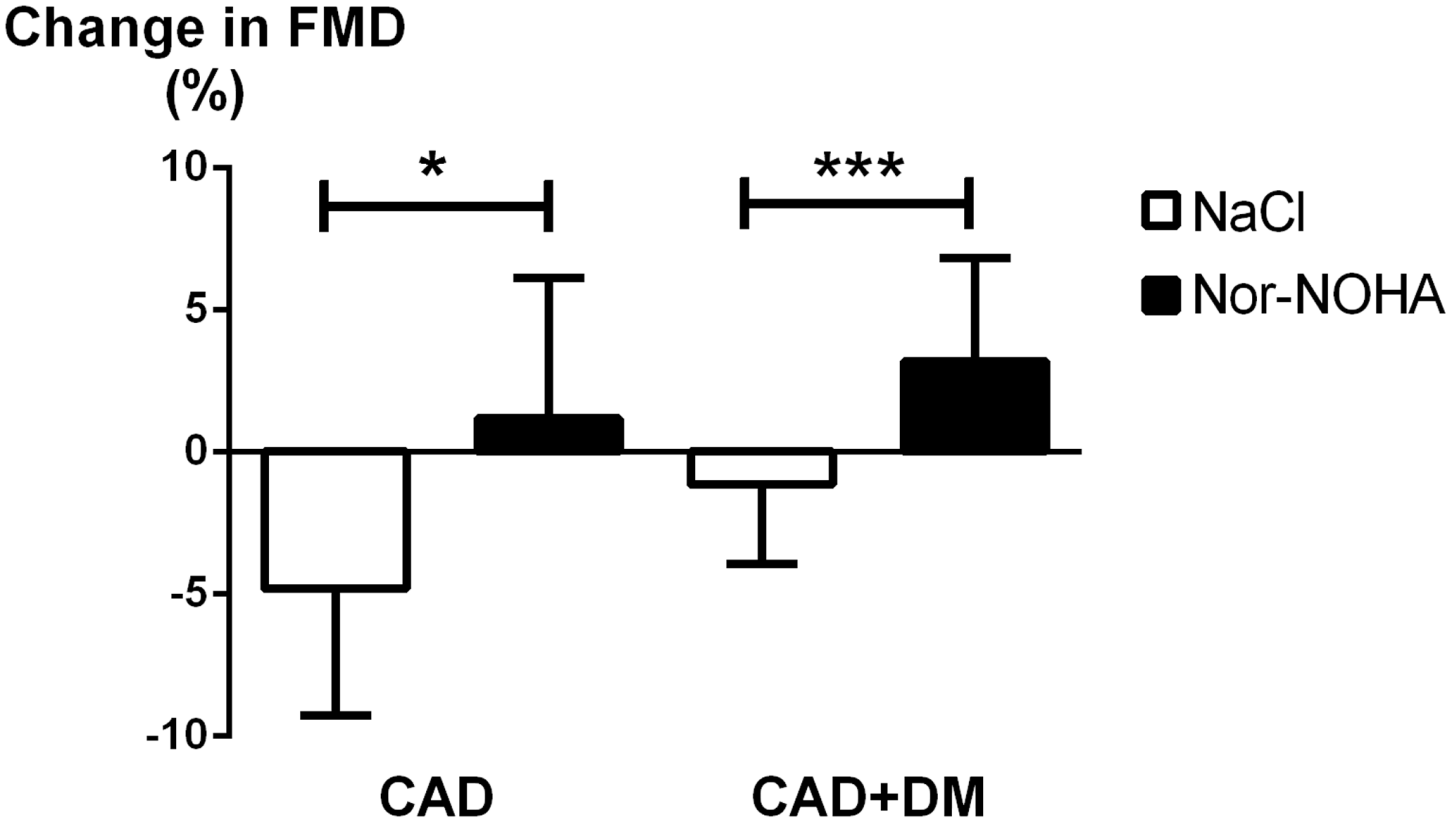
Response to ischemia-reperfusion. Change in flow-mediated dilatation (FMD) from baseline induced by ischemia-reperfusion in patients with coronary artery disease (CAD) and CAD plus type 2 diabetes mellitus (CAD+DM) given saline or the arginase inhibitor N^ω^-hydroxy-nor-arginine (nor-NOHA). Data are presented as means and SD; *P<0.05, ***P<0.001.

Baseline endothelium-dependent vasodilatation determined by FMD did not differ between the study occasions in any of the patient groups (Fig. 2). In the CAD group FMD was reduced by 4.8% (P<0.05) following ischemia-reperfusion during administration of saline. Administration of the arginase inhibitor nor-NOHA completely prevented the decrease in FMD induced by ischemia-reperfusion (Fig. 2). FMD in the CAD group after ischemia-reperfusion was significantly greater following administration of nor-NOHA than following administration of saline (Fig. 3).

Baseline endothelium-dependent vasodilatation was significantly lower in the CAD+DM group than in the CAD group (P<0.05). Ischemia-reperfusion did not induce a significant reduction in FMD in the CAD+DM group (Fig. 2). By contrast, arginase inhibition improved FMD after ischemia-reperfusion in the CAD+DM group (Fig. 2). Thus, following ischemia-reperfusion FMD was significantly greater after administration of nor-NOHA than after saline administration (Fig. 3). The improvement in FMD by arginase inhibition following ischemia-reperfusion was 3.6% in the CAD group and 4.0% in the CAD+DM group (P = 0.49; Fig. 2). When all patients (CAD and CAD+DM) were analyzed together, the FMD was significantly reduced following ischemia-reperfusion after saline administration (P<0.01). By contrast, FMD was not attenuated by ischemia-reperfusion after administration of nor-NOHA.

Endothelium-independent vasodilatation induced by sublingual nitroglycerine did not differ between groups or between interventions. In the CAD group endothelium-independent vasodilatation was 11.8±4.1% following saline and 14.3±5.0% following nor-NOHA administration (P = 0.18). In the CAD+DM group endothelium-independent vasodilatation was 13.6±6.4% following saline and 12.3±6.7% following nor-NOHA administration (P = 0.51).

### Radial artery diameter

Baseline diameters of the radial artery during the experimental protocols are presented in Table 2. There was no difference in radial artery diameter between the two interventions in either the CAD group the CAD+DM group.

**Table 2:** Radial artery diameter.

**CAD (NaCl)**		**CAD (Nor-NOHA)**	
Before ischemia	After ischemia	Before ischemia	After ischemia
2.81±0.36	2.91±0.37	2.90±0.26	2.93±0.37
**CAD+DM (NaCl)**		**CAD+DM (Nor-NOHA)**	
Before ischemia	After ischemia	Before ischemia	After ischemia
2.77±0.36	2.81±0.36	2.94±0.37	2.91±0.36

Data (mean and SD) are baseline radial artery diameter in mm before and after ischemia-reperfusion in the CAD and in the CAD+DM groups randomized to during either saline or nor-NOHA infusion. CAD, coronary artery disease. DM, type 2 diabetes mellitus. NaCl, saline. Nor-NOHA, N^ω^-hydroxy-nor-arginine.

## Discussion

The main finding of the current study is that administration of an arginase inhibitor as compared to vehicle improves endothelium-dependent vasodilatation following ischemia-reperfusion in patients with CAD. These data suggests that arginase plays an important role in the pathophysiology of ischemia-reperfusion injury in patients with CAD, and that inhibition of arginase provides a promising therapeutic strategy to prevent endothelial injury induced by ischemia-reperfusion.

Emerging data suggest that up-regulation of arginase is a critical factor behind reduced bioavailability of NO by its competition with eNOS for their common substrate L-arginine [10]. Interestingly, arginase expression is rapidly increased and NO production is reduced following myocardial ischemia-reperfusion [11], [14]. A prominent up-regulation of arginase activity is observed already 20 min after reperfusion in rats subjected to 30 min of coronary artery ligation and reperfusion without an increase in protein expression [14]. The functional effect of arginase inhibition has been evaluated in experimental ischemia-reperfusion studies [13], [14], [25]. Endothelium-dependent vasorelaxation of pig coronary arterioles was restored by an arginase inhibitor [13]. Of additional importance, arginase inhibition reduced infarct size by approximately 50% in a rat and pig model of myocardial ischemia-reperfusion [12], [13]. This reduction in infarct size was abolished by a NOS inhibitor suggesting the effect was mediated by NO production. Furthermore, lack of the substrate for NOS as a result of arginase up-regulation leads to a process called NOS uncoupling, i.e. when the enzyme produces reactive oxygen species [9]. By inhibiting arginase, more substrate is available for NOS, which reduces the uncoupling process [26], [27] and improves endothelial function. The present study was therefore undertaken to investigate whether arginase inhibition protects from ischemia-reperfusion injury in patients with CAD. The arginase inhibitor nor-NOHA, administered shortly before and during the early part of reperfusion, completely prevented the impairment in endothelium-dependent vasodilatation induced by ischemia-reperfusion in patients with CAD. This observation demonstrates that arginase is of importance for the development of ischemia-reperfusion injury in humans and that inhibition of arginase may be used to prevent ischemia-reperfusion injury.

One interesting finding was that ischemia-reperfusion *per se* did not induce a significant impairment in endothelial function among patients with CAD+DM. The exact reason for this phenomenon is unclear. One explanation may be that the patients with CAD+DM had lower baseline FMD in comparison with those with CAD only. Possibly, 20 min ischemia and reperfusion may not induce significant further reduction of endothelial function in this patient group. The importance of arginase for endothelial dysfunction in patients with CAD+DM was demonstrated by the observation that FMD following ischemia-reperfusion was significantly greater after administration of nor-NOHA than after saline administration. Thus, the net increase in FMD by arginase inhibition following ischemia-reperfusion was similar in the CAD and the CAD+DM groups. Collectively these observations suggest that arginase inhibition improved endothelial function following ischemia-reperfusion among patients with CAD with and without type 2 diabetes.

We used the forearm model to extend previous knowledge regarding the role of arginase in experimental models to explore the role of arginase for development of ischemia-reperfusion injury in humans. The model is suitable for interventional studies evaluating endothelial function in humans and patients with co-morbidities. Since co-morbidities have been suggested to importantly influence the effect of interventions targeting ischemia-reperfusion injury [16] the efficacy of therapeutic strategies are needed to be tested in patients with atherosclerosis, diabetes and other co-morbidities. Although endothelial function is a surrogate end point for ischemia-reperfusion injury, endothelial cell damage is one early and important component of the reperfusion injury cascade. FMD correlates with coronary endothelium-dependent vasodilatation [28] and has been shown to predict future cardiovascular events [29], which makes it a relevant model for the present type of study. It is therefore of potential clinical importance that inhibition of arginase protects from endothelial dysfunction induced by ischemia-reperfusion in patients with CAD. Although the endpoint of the study is endothelial function, the data clearly suggest an important therapeutic effect of arginase inhibition in the setting of ischemia-reperfusion injury. If this observation is applicable for the prevention of reperfusion injury in ST-elevation myocardial infarction or following coronary artery by-pass surgery remains to be established.

Endothelium-independent vasodilatation induced by nitroglycerine administration did not differ between groups or visits indicating that it was not affected by arginase inhibition. The lack of effect on nitroglycerine-induced dilatation suggests that the beneficial effect of arginase inhibition on FMD following ischemia-reperfusion was mediated via endothelium-derived NO.

There are certain limitations with this study. The study was performed on a limited number of patients. However, the power calculation was based on an improvement in FMD by the intervention by 3%, which was reached in both groups. The study was not double-blind. On the other hand, the operator performing the measurements and evaluation of the results was blinded to the randomization of intervention. FMD was performed on the radial artery since the brachial artery was catheterized for infusion of substances. Velocity measurement was not determined since it would have compromised the continuous high quality recording of the artery diameter. However, we know from a previous study that arginase inhibition does not change basal forearm blood flow [15]. The place of measurement was distal to the position blood pressure cuff used to induce the ischemia and hyperemia for the baseline FMD. Thus, the area of measurement was subjected to one cycle of five min ischemia before the index ischemia, which may limit the reduction in FMD due to local preconditioning [19]. However, in the CAD group a clear decrease in FMD after index ischemia was observed. Furthermore, if any preconditioning effect occurred, it would have underestimated the beneficial effect of arginase inhibition.

In conclusion, the present study demonstrates that the arginase inhibition protects from endothelial dysfunction induced by ischemia-reperfusion in patients with CAD. These data suggests that arginase plays an important role in the pathophysiology of ischemia-reperfusion injury, and provide further evidence that it is a critical regulator of endothelial function in humans with cardiovascular disease. The results suggest that arginase inhibition may be a promising therapeutic strategy to limit ischemia-reperfusion injury in patients with CAD and further studies in patients with acute myocardial infarction are therefore warranted.
